# How does the rise in contraceptive usage predict pregnancy termination among young women in Kenya? an in-depth multilevel analysis

**DOI:** 10.1186/s12889-025-21580-3

**Published:** 2025-02-07

**Authors:** Stephen Okechukwu Chukwudeh, Obasanjo Afolabi Bolarinwa, Oluwatobi Abel Alawode, David Olawade, Sinegugu Shongwe, Ritika Tiwari

**Affiliations:** 1 Department of Criminology and Security Studies, Federal University Oye-Ekiti, Oye-Ekiti, Ekiti Nigeria; 2 Department of Public Health, School of Business and Health Studies, York St John University, London, United Kingdom; 3 Demography and Population Studies Programme, Schools of Public Health and Social Sciences, University of the Witwatersrand, Johannesburg, South Africa; 4 Department of Sociology and Criminology & Law, University of Florida, Gainesville, FL USA; 5 Department of Public Health, University of East London, London, United Kingdom; 6 Department of Public Health, University of KwaZulu-Natal, Durban, South Africa

**Keywords:** Contraceptive use, Pregnancy termination, Pregnancy history, Unwanted pregnancy, Young women

## Abstract

**Background:**

Young women were less likely to practise consistent contraceptive use and are also known to exhibit risky sexual behaviours, which could lead to unintended pregnancy. Studies have also shown that about half of the Kenyan population is young, an age bracket that predominantly experiences unwanted pregnancy. However, adequate attention has not been given to the relationship between pregnancy termination and contraceptive use among this teeming population in Kenya. Thus, this study examined the association between pregnancy termination history and contraceptive use among women aged 15–24 years old in Kenya.

**Methods:**

A total of 12,166 women aged 15–24 years old from Kenya were drawn using a secondary dataset from the most recent Kenya Demographic Health Surveys conducted in the year 2022. Frequency distribution and multilevel logistic regression analyses were employed to determine the association between contraceptive use and pregnancy termination history among young women aged 15–24 years old with covariates at *p* < 0.05.

**Results:**

The results showed that the higher percentage of contraceptive users among young women are those aged 15–19 years (50.1%); consequently, a higher percentage of pregnancy termination was also found among women who reported the use of contraceptive methods (6.2%) compared to 2.9% among non-users. The main findings of the study showed that young women who reported using contraceptives were more likely to have a history of pregnancy termination [aOR = 1.03; 95% CI: 0.80–1.26] compared to those who were non-users. Also, age, marital status, and parity are significantly associated with the history of pregnancy termination.

**Conclusion:**

Our analyses established an association between history of contraceptive use and termination of pregnancy among young women between 20 and 24 years in Kenya. Place of residence, age, wealth index, level of education, and met needs of children have been identified as risk factors. Intervention to reduce pregnancy termination in Kenya should focus on women aged 20–24 years, those with no formal education, urban residence, and women with met need for children.

## Background

Africa has a higher continental average of younger individuals than the global population, and the United Nations estimates that about 70% of the sub-Saharan African (SSA) population is less than 30 years of age [1]. Within Kenya, individuals between the ages of 15 and 24, specifically women, make up approximately 15% of the total population of 47 million, translating to an estimated 7 million [2].

With a total fertility rate (TFR) of 3.4, which is higher than the global average of 2.27 [3, 4], this shows that young women in Kenyan are highly fecund and are particularly at risk of pregnancies, with 47% of such pregnancies being unwanted, according to the Kenya Demographic and Health Survey (KDHS) [5]. In addition, evidence has shown that young individuals exhibit risky sexual behaviours [6], with the same study revealing that 59.4% of first-year university students in Kenya engaged in sexual activity, with many having their first sexual experience at a young age (< 20 years). However, only 32.5% reported consistent practice of condom use [6].

Consequently, a cross-level countries study conducted by Sedgh et al. [7] in 2014 shows that the national teenage abortion rate in Kenya was 38 per 1000 girls aged 15–18, representing one of the highest in the world [7]. This is coupled with a highly prevalent adolescent first birth rate [8], even though adolescent motherhood comes with a lot of negative ramifications for girls and their newborns [9].

Another recent finding in Kenya showed that the majority of these adolescent girls and young women [AGYW] are aware of the dangers of unwanted pregnancy, including social shame, partner rejection, and disruption of their schooling [10, 11]. Also, they are aware of the different forms of birth control available but hold several misconceptions regarding these commodities [12], which affects their decision not to use various methods of contraception.

Furthermore, complicating the situation is the discrimination and stigma faced by these young individuals in accessing contraceptive care, even when they are willing and enlightened to do so [13]. The resultant effect is usually unplanned pregnancy, and many of them turn to unsafe abortion practices, which lead to 2600 deaths a year [14]. Notwithstanding, the Kenyan government has been making considerable efforts to improve accessibility to contraceptive services, as reported by the Kenya Service Provision Assessment that about 90% of service providers offer them [15].

Abortion laws are also becoming flexible as a result of substantial changes since 2010 in Kenya [16], in part because health is recognised as a fundamental right in the country’s constitution. However, the 1963 Penal Code still makes abortion illegal, which puts women and girls seeking care at risk of intimidation, false accusations, and legal repercussions [17]. Nevertheless, the Kenyan High Court upheld abortion as a fundamental right in 2022 and ruled that it is unlawful to arrest and prosecute anyone who has or provides an abortion [18].

AGYW in Kenya experiences one of the highest teenage abortion rates globally [18] and even abortion rates generally [19], and various factors have been associated with pregnancy termination in the country [12, 13, 20, 21]. Many of these findings have centred around demographic and socioeconomic factors, parity status, and type of contraceptive method, especially for the full spectrum of women of reproductive ages [12, 13, 21]. Even though the literature in the geographical context of the study is filled with evidence on pregnancy termination, these studies have several shortcomings.

Most importantly, there is a conspicuous lack of studies at the national level exploring pregnancy termination, particularly as it relates to AGYW. Additionally, given the rising prevalence of contraceptive use in the country, there hasn’t been an attempt to explore how this is associated with the likelihood of pregnancy termination among this population group. Thus, this present study attempts to fill this gap in the literature.

The connection between contraceptive use and pregnancy termination is often seen as potentially confusing, particularly when both show an increase simultaneously, especially in specific populations, contrary to conventional expectations [22]. In societies like this, individuals perceive that their risk of unwanted pregnancy is low [23]; this is because the emergence of modern contraception, in particular, is associated with a destabilisation of high fertility preferences, especially in places experiencing fertility transition. Therefore, with the increase in contraceptive prevalence and the decline in fertility, a growing percentage of couples express a desire for fewer children or a significant delay before the next child. Consequently, there is a heightened exposure to the risk of unintended pregnancy, especially if there is no intentional effort to avoid unplanned pregnancy [24]. Given this perspective, this study fills the gap in the literature by exploring the association between the contraceptive use experience of AGYW and their history of pregnancy termination. By addressing these issues, this study contributes to a deeper understanding of the intricate relationship between contraceptive use and pregnancy termination of young Kenyan women in the face of a plethora of initiatives on the reproductive choices and outcomes of young women while also considering the recent legal developments around abortion as a fundamental right. The findings will inform policies and interventions aimed at enhancing reproductive rights and outcomes for this vulnerable demographic, ultimately contributing to their well-being and the broader public health landscape.

## Data and methods

### Study design and participants

This cross-sectional study extracted and analysed data from the 2022 KDHS. The KDHS is a national survey (implemented by the Kenya National Bureau of Statistics (KNBS) and Ministry of Health (MoH)) that collected data from respondents on their socio-demographic characteristics, maternal and child health, and other sexual and reproductive health-related indicators such as pregnancy termination, contraception, family planning, fertility, intimate partner violence, etc., from all women of reproductive age group using a questionnaire. However, our study focused on young women aged 15–24 years of age at the time of the survey who responded to the questions on pregnancy termination history, and this resulted in an analytical sample of 12,166 young women [5, 10, 21].

The KDHS employed a two-staged cluster design sampling technique to select the primary sampling unit from which enumeration was achieved [5]. The second stage was the listing of households to select enumeration areas to derive a representative sample for the country. More information about the study design and survey instruments can be found here [5]. The DHS datasets employed in this study are publicly available on the Demographic and Health Survey (DHS) website and can be downloaded for free upon request via https://dhsprogram.com/data/available-datasets.cfm.

### Study variables

#### Outcome variable

The outcome variable is a self-reported history of pregnancy termination; in the survey, the women were asked whether they have ever had a pregnancy terminated, and the responses were coded “Yes = 1” if the respondent had ever terminated pregnancy and “No = 0” if the respondent had never terminated pregnancy.

#### Explanatory variables

The explanatory variable in this study is the correspondents’ contraceptive use history. The response of the women to questions on their contraceptive use history, i.e., whether they have ever used anything to avoid getting pregnant. The responses included “not using” and “use” of various short, long-acting, and permanent methods. For the study, we measured contraceptive use as “Yes = 1” for those who have ever used any method to avoid getting pregnant, while those who reported never using a method were coded as “No = 0”.

#### Covariates

Covariates for this study were selected based on variables that showed association at the bivariate levels and other similar studies in the literature [25, 26]. These include age, age at first marriage, women’s highest level of education, partner’s highest level of education, type of place of residence, parity, type of marriage, work status, level of exposure to mass media, household wealth index, and the sex of the household head. The ages of the young women were coded as 15–19 & 20–24, while the highest level of education was a categorical variable– no education, primary, secondary, and higher. We categorised marital status into never married, married/cohabiting, and previously married. Parity was also included as a covariate with categories ranging from one child to four or more; the household wealth index was used as conceptualised in the DHS survey (Poorest, Poorer, Middle, Richer & Richest). At the household level, we included the sex of the household head with two categories: male and female. Type of place of residence variable: rural and urban, while for community-level socioeconomic disadvantage, we developed textiles ranging from least disadvantaged to most disadvantaged based on the individual socioeconomic measure.

### Statistical analyses

The data analysis was conducted at three levels: univariate, bivariate, and multivariate. At the univariate level, frequency and percentage distributions of all the study variables were reported. At the bivariate level, a chi-square test was used to assess the association between contraceptive use and the history of pregnancy termination, also providing the distribution of pregnancy termination history across the main explanatory variable and covariates.

For the multivariate analysis, a multilevel regression modelling with mixed and random effects was employed due to its ability to handle hierarchical data structures and account for multiple levels of influence on the outcome variable [27, 28]. Five models were fitted, including the null model (Model 0), which assessed variations in pregnancy termination history across communities without adjusting for explanatory variables or covariates. Model 1 estimated the association between contraceptive use and pregnancy termination history, while Model 2 introduced covariates. Model 3 included contraceptive use status, household-level, and community-level variables. The final model (Model 4) accounted for the main explanatory variable along with individual, household, and community-level covariates.

The fixed effects of the models were reported using adjusted Odds Ratios (aOR) with 95% confidence intervals. Random effects were evaluated using the Intra-Cluster Correlation Coefficient (ICC) [27], which quantifies the proportion of total variance in pregnancy termination attributable to community-level clustering [28]. The ICC was calculated as the ratio of variance of interest (community-level) to the total variance (variance of interest plus residual variance), indicating the extent of clustering in the data. Higher ICC values suggest substantial clustering effects, underscoring the importance of multilevel modelling in studies with community-based characteristics [29].

To account for potential biases, survey sample weights were applied to correct for non-response and under-sampling, while missing values were excluded from the analysis. All statistical analyses were performed using Stata version 14.1 [30]. These methodological considerations ensure robustness and reliability in addressing the study objectives.

## Result

Table 1 shows the distribution of study variables and the percentage of young women reporting a history of pregnancy termination by explanatory variables. It can be reported that 29% of young women reported contraceptive use, and more than half are between the age group of 15–19 [50.1%], 56% of the young women have secondary education, 71% have never married while 66% do not have a child. For the household wealth index, 23% and 17% are from the richest and poorest households, respectively; the analysis also showed that 61% of the young women reside in rural areas while 62% are from male-headed households, and 48% are from the least socioeconomically disadvantaged communities. The distribution of the history of pregnancy termination by contraceptive use history and other covariates was also shown in Table 1: 3% (3%) of young women who are not using contraceptives and have ever terminated a pregnancy compared to 6% among those using contraceptives. The study also found that the highest percentage of young women with a history of pregnancy termination is among those with no education (8%), those currently married/cohabiting (10%), those with four or more children (11%), those residing in urban areas (5%), and those residing in socioeconomically disadvantaged neighbourhoods (4%).

**Table 1:** Background characteristics and proportion of pregnancy termination history

*N* = 12,166	% [*n*]	History of pregnancy termination		*P*-Value
		No	Yes	
**Contraceptive use**				0.000
Not Using	71.3 [8674]	8821 [97.1]	262 [2.9]	
Using	28.7 [3492]	2892 [93.8]	191 [6.2]	
**Age groups**				0.000
15–19	50.1 [6095]	6334 [98.9]	70 [1.1]	
20–24	49.9 [6071]	5379 [93.4]	383 [6.7]	
**Highest level of education**				0.000
No Education	2.5 [304]	730 [92.5]	59 [7.5]	
Primary	26 [3163]	3351 [95.1]	173 [4.9]	
Secondary	55.6 [6764]	6298 [97.3]	172 [2.7]	
Higher	15.8 [1922]	1334 [96.5]	49 [3.5]	
**Marital status**				0.000
Never Married	70.9 [8626]	8316 [99.2]	65 [0.8]	
Married/Cohabiting	26.1 [3175]	3004 [89.6]	350 [10.4]	
Previously Married	3.0 [365]	393 [91.2]	38 [8.8]	
**Parity**				0.000
None	65.8 [8005]	7707 [98.3]	134 [1.7]	
One	22.9 [2786]	2572 [93.6]	175 [6.4]	
Two	8.7 [1058]	1018 [91.0]	101 [9.0]	
Three	2.1 [255]	317 [91.1]	31 [8.9]	
Four or more	0.6 [73]	99 [89.2]	12 [10.8]	
**Household wealth index**				0.005
Poorest	17.2 [2093]	2686 [95.5]	126 [4.5]	
Poorer	19.6 [2385]	2277 [96.9]	74 [3.2]	
Middle	19.1 [2324]	2372 [97.3]	67 [2.8]	
Richer	21.6 [2628]	2536 [95.8]	111 [4.2]	
Richest	22.5 [2737]	1842 [96.1]	75 [3.9]	
**Type of place of residence**				0.001
Urban	38.8 [4720]	4374 [95.5]	205 [4.5]	
Rural	61.2 [7446]	7339 [96.7]	248 [3.3]	
**Household head sex**				0.000
Male	61.6 [7494]	7191 [95.6]	328 [4.7]	
Female	38.4 [4672]	4522 [97.3]	125 [2.7]	
**Socioeconomic disadvantage**				0.001
Tertile 1 [Least Disadvantage]	47.8 [5815]	6602 [95.8]	290 [4.2]	
Tertile 2	11.9 [1448]	1402 [97.0]	43 [3.0]	
Tertile 3 [Most Disadvantage]	40.3 [4903]	3709 [96.9]	120 [3.1]	

### Fixed effects (measures of association) results

In Table 2, Model 4 is the complete model that shows the association between contraceptive uses, individual and contextual level factors, and pregnancy termination among young women in Kenya. At the individual level, contraceptive use, age, highest level of education, marital status, and parity were associated with pregnancy termination. In contrast, none of the community-level variables showed an association with pregnancy termination. However, household wealth, type of place of residence, and sex of household head showed association in model 3. The unadjusted model shows that young women who reported using contraceptives are more than two times more likely to report a history of pregnancy termination compared to non-users of contraceptives [aOR = 2.26; 95% CI: 1.86–2.75], and similar results showed up in Model 3 when we added household and community level variables, young women who reported using contraceptives are more than two times more likely to report a history of pregnancy termination compared to non-users of contraceptives [aOR = 2.27; 95% CI: 1.86–2.76].

In the complete model, it was found that young women who are using contraceptives are 3% more likely to terminate pregnancy compared to those who are not using [aOR = 1.03; 95% CI: 0.80–1.26]. It was also found that young women aged 20–24 are more than two times significantly more likely to have terminated a pregnancy compared to those aged 15–19 [aOR = 2.69; 95% CI: 1.97–3.67]. Furthermore, the analysis showed that young women with secondary and higher education are 34% and 46% significantly less likely to have terminated a pregnancy, respectively, compared to young women with no education. Married/Cohabiting and previously married young women are more than 13 times [aOR = 13.11; 95% CI: 9.29–18.50] and 10 times [aOR = 10.15; 95% CI: 6.31–16.33]. More likely to terminate a pregnancy compared to those who have never been married. It was also found that higher parity is significantly associated with a lower likelihood of pregnancy termination compared to young women with zero parity.

### Random effects [measures of variation] results

The empty model [Model 0] revealed variation in the probability of pregnancy termination concerning the clustering of PSUs [σ2 = 0.32, 95% CI 0.14–0.75]. The empty model indicated that 9% of the overall variance in pregnancy variation is attributable to inter-cluster variation in the characteristics [ICC = 0.09]. For model 1, the probability of pregnancy termination did not vary (σ2 = 0.33, 95% CI 0.14–0.77). However, in model 3, there was a drop in the overall variance in pregnancy termination attributable to inter-cluster variation of the characteristics (5%). This indicates that the variation in pregnancy termination is highly attributable to differences or variations in factors at the community level, as shown in Model 2. In Model 3, the ICC increased to 7% while it dropped to 5% in the full model, and there was a drop in variation in the probability of pregnancy termination concerning the clustering of PSUs [σ2 = 0.18, 95% CI 0.04–0.85].

**Table 2:** Multilevel logistic regression of the relationship between contraceptive use and history of pregnancy termination among young women in Kenya

*N* = 12,166	Model 0	Model 1 OR [95% CI]	Model 2 aOR [95% CI]	Model 3 aOR [95% CI]	Model 4 aOR [95% CI]
**History of pregnancy termination**			**Odds Ratio**		**Odds Ratio**
**Contraceptive use**					
Not Using					
Using		2.26 *** [1.86–2.75]	1.01 [0.81–1.27]	2.27 ** [1.86–2.76]	1.03* [0.80–1.26]
**Age**					
15–19					
20–24			2.82 *** [2.07–3.84]		2.69 *** [1.97–3.67]
**Highest level of education**					
None					
Primary			1.07 [0.76–1.50]		1.07 [0.75–1.51]
Secondary			0.68 ** [0.48–0.97]		0.66 ** [0.45–0.96]
Higher			0.62 ** [0.39–0.98]		0.54 ** [0.33–0.89]
**Marital status**					
Never Married					
Married/Cohabiting			13.11 *** [9.36–18.36]		13.11 *** [9.29–18.50]
Previously Married			10.52 *** [6.55–16.89]		10.15 *** [6.31–16.33]
**Parity**					
Zero					
One			0.62 ** [0.46–0.82]		0.65 ** [0.48–0.87]
Two			0.52 *** [0.37–0.73]		0.56 ** [0.39–0.79]
Three			0.41 *** [0.26–0.66]		0.45 ** [0.28–0.73]
Four or more			0.50 ** [0.25–0.99]		0.55 [0.28–1.10]
**Household wealth index**					
Poorest					
Poorer				0.67 ** [0.49–0.90]	0.99 [0.71–1.38]
Middle				0.54 *** [0.39–0.75]	0.83 [0.58–1.20]
Richer				0.66 **[0.47–0.94]	1.02 [0.68–1.52]
Richest				0.57 ** [0.37–0.87]	1.17 [0.72 - 1.89]
**Residence**					
Urban					
Rural				0.62 ** [0.46–0.83]	0.75 [0.55–1.02]
**Household head sex**					
Male					
Female				0.61 *** [0.49–0.75]	1.07 [0.85–1.36]
**Socioeconomic Disadvantage**					
Tertile 1 [Least Disadvantage]					
Tertile 2				0.74 [0.52–1.05]	0.89 [0.62–1.27]
Tertile 3 [Most Disadvantage]				0.83 [0.64–1.08]	1.03 [0.77–1.32]
**Random effect result**					
PSU Variance [95% CI]	0.32 [0.14–0.75]	0.33 [0.14–0.77]	0.19 [0.04–0.83]	0.25 [0.09–0.73]	0.18 [0.04–0.85]
ICC	0.09	0.09	0.05	0.07	0.05
LR Test	𝑥2 = 6.27; *p* = 0.0062	𝑥2 = 6.20; *p* = 0.0064	𝑥2 = 1.90; *p* = 0.0839	𝑥2 = 3.80; *p* = 0.0256	𝑥2 = 1.76; *p* = 0.0923
Wald chi-square		66.41	428.45	118.9	435.4
**Model Fitness**					
Log-likelihood	-1931.9	-1900.3	-1599.5	-1871.7	-1592.8
BIC	3882.7	3828.8	3349.6	3846.9	3411.3
AIC	3867.9	3806.6	3231.1	3765.4	3233.6
n	12,166	12,166	12,166	12,166	12,2166

CI = Confidence Interval; cOR = Crude Odds Ratio; aOR = Adjusted Odds Ratio; ref = Reference; *=*p* < 0.05; **=*p* < 0.01; ***=*p* < 0.001

Model 0 is the empty column that reports the base random effect result

Model 1 showed the uncontrolled or unadjusted association between contraceptive use and pregnancy termination history

Model 2 controlled for covariates such as age, education, age at first marriage, marital status and parity

Model 3 controlled for covariates such as household wealth index, residence, household head sex and socioeconomic disadvantage

Model 4 controlled for all the explanatory variables such as age, education, age at first marriage, marital status, parity, household wealth index, residence, household head sex, and socioeconomic disadvantage

## Discussion

The lack of contextual literature on the association between contraceptive use and pregnancy termination among young women in Kenya motivates this study, and we sought to establish a connection among young women in the context of Kenya– where the abortion death rate is significantly high.

The results from the study show that there is a higher frequency of pregnancy termination among young women aged 20–24. This high prevalence implies that this group of young women have a higher likelihood of pregnancy termination compared to women aged 15–19. This confirms what previous studies have found [31, 32] about the low prevalence of contraceptive use among young people and the higher pregnancy termination rate among young women 20–24 years old in Kenya.

We also found an association between contraceptive use and a history of pregnancy termination; specifically, young women who reported using contraceptives have a higher likelihood of pregnancy termination, which sort of appears counterintuitive, but as earlier explained, such a parallel relationship between contraceptive use and pregnancy termination is a possibility in populations undergoing transition or even pre-transition societies like Kenya, where individuals perceive that their risk of unwanted pregnancy is low [33, 34].

The association between contraceptive use and a history of pregnancy termination could be better contextualised within Kenya’s demographic landscape because while fertility rates in Kenya have been steadily declining since 2022, teenage pregnancy rates remain relatively high, with varied low geographical median age for first birth [5]. This dynamic reflects a population in transition, where declining fertility coexists with traditional norms and behaviours [5]. In such contexts as this, young women may perceive a low risk of unwanted pregnancy, leading to inconsistent or incorrect contraceptive use, which could contribute to higher rates of pregnancy termination [31].

Consequently, the emergence of modern contraception, in particular, is associated with a destabilisation of high fertility preferences, especially in places experiencing fertility transition. Thus, as contraceptive prevalence rises and fertility starts to fall, an increasing proportion of couples want no more children (or want an appreciable delay before the next child), and exposure to the risk of unintended pregnancy also increases as a result [35]. Additionally, contraceptive failure or inconsistent use may be key factors contributing to the high rates of pregnancy termination observed in this population [31].

Furthermore, evidence from Kenya has reported a high sexual activity and non-use of contraceptives among young people, especially young women 20–24 years of age [27], which has warranted efforts at increasing the prevalence of contraceptive use among young people in the country and other countries in SSA [36]. This finding implies that such efforts haven’t been achieving the right goals of reducing unintended pregnancy among young women in Kenya. Previous studies have reported similar findings on the relationship between contraceptive use and history of pregnancy termination. For instance, a multi-country study in SSA [37, 38] found that women whose Contraceptive needs have been met are more likely to report pregnancy termination. Just as we have mentioned, this relationship between contraceptive use and termination of pregnancy seems counterintuitive, but explanations that have also been offered centre around contraceptive failure, which is even stronger among women under the age of 30 [38].

Cleland [39], in a study explaining the complex relationship between contraception and abortion, opined that accidental pregnancy while using a method is a possibility in about 30% of all unintended pregnancies, and this can be explained by the fact that the likelihood of unintended pregnancy increases when desired family sizes decrease– which evidence has shown to be happening in Kenya, as fewer reproductive years are dedicated to planned pregnancies and the rise in contraceptive usage in recent years has lessened the risk of unintended pregnancies. This impact has been tempered in various global regions due to a growing tendency to terminate such pregnancies [39–41].

On this basis, the implications of a high prevalence of sexual intercourse without the use of contraceptive methods are evident, especially among young women who could also face the consequences of their actions, such as unsafe abortion [42], school drop-out [43], poor child spacing [44], intergenerational poverty [45], and preterm delivery [46].

Beyond contraceptive use, other covariates along these themes of demographic and socioeconomic factors showed an association with the likelihood of pregnancy termination. For instance, young women with low levels of education have a higher likelihood of pregnancy termination experience, and married young women or even those who reside in urban areas exhibit a higher likelihood of pregnancy termination. These findings are consistent with what has been reported in previous studies among adolescent girls and young women in SSA [47, 48].

Our study set out to explain the association between contraceptive use and the history of pregnancy termination among adolescent girls and young women in Kenya. An association has been established which confirms the counterintuitive argument in the literature. Hence, future studies in this context must explore the dynamics that play into this relationship. However, another plausible explanation could be that pregnancy termination may precede contraceptive use [49]. Women who have experienced a termination may be more likely to use contraception afterwards, either due to counselling or being prescribed contraceptives post-termination to prevent future unintended pregnancies [49]. This aligns with findings from other studies showing that women who have given birth are often more likely to use contraception, as postpartum contraceptive counselling and prescriptions are standard practice in many healthcare settings [50, 51]. Thus, the apparent association between contraceptive use and pregnancy termination may reflect a sequence of events rather than a direct relationship.

### Strengths and limitations

This study benefits from the use of recent and reliable data derived from a well-representative, large sample size in Kenya, allowing the findings to be generalised to young women regarding contraceptive use and pregnancy termination within the country. However, due to the cross-sectional design of the KDHS data utilised, causal relationships cannot be established, as no longitudinal analysis was conducted.

Additionally, some important variables and time-related information that could provide a more comprehensive understanding of the causes of pregnancy termination among this population were unavailable in the KDHS dataset. Furthermore, the self-reported nature of pregnancy termination data may introduce recall bias, potentially leading to either under- or overestimation. Stigma and fear associated with disclosing pregnancy termination could also contribute to underreporting.

Finally, it is essential to highlight that the DHS definition of pregnancy termination encompasses stillbirths and miscarriages, which may not align with the respondents’ intentions, potentially impacting the interpretation of the findings.

### Implication for research and policy

Factors associated with the history of contraceptive use and pregnancy termination, as identified in this study, will help health practitioners and policymakers to make informed decisions as regards proper family planning, especially for young women who have met their desired number of children. This is germane to avoid the risk associated with the process of terminating pregnancy as well as avert both long and short-term consequences of unintended pregnancy and unsafe termination of their pregnancy. This will help develop targeted intervention strategies for women aged 20–24 years who are more prone to pregnancy termination due to low levels of education, place of residence, and low awareness of using contraception.

### Conclusion and recommendations

Our analysis established association between contraceptive use history and pregnancy terminations remains prevalent among young women in Kenya, particularly among young women aged 20–24 years. Place of residence, age, wealth index, education level, and met needs of children have been identified as key risk factors. Interventions to reduce pregnancy termination in Kenya should prioritise women aged 20–24, those with no formal education, urban residents, and women with met needs for children.

This study adds to the limited literature by examining the association between contraceptive use and pregnancy termination among young women in Kenya, where unsafe abortion rates are alarmingly high. Our findings indicate that young women who report using contraceptives are more likely to have experienced pregnancy termination. While this may appear counterintuitive, it reflects a broader demographic transition in Kenya. As fertility rates decline and contraceptive prevalence increases, more women are exposed to risks of unintended pregnancy due to contraceptive failure or inconsistent use, especially in populations that perceive a low risk of unwanted pregnancy. This dynamic is further complicated by high sexual activity and low contraceptive use among adolescents and young adults, implying that existing efforts to increase contraceptive prevalence may not adequately address unintended pregnancies among this group.

Additionally, women with low education levels, urban residence, and marital status exhibited a higher likelihood of pregnancy termination, consistent with previous studies in sub-Saharan Africa. These findings underscore the importance of targeted interventions that consider socio-demographic factors, education campaigns, and improved access to high-quality contraceptive methods to reduce contraceptive failure rates.

Finally, we emphasise the need for future research to explore the dynamics between contraceptive use and pregnancy termination in greater depth by conducting a qualitative study or longitudinal study. Understanding these dynamics will enable the design of more effective strategies to address the complex relationship between contraception and pregnancy termination and ultimately reduce unsafe abortions and their consequences among young women in Kenya.

## Data Availability

The datasets utilised in this study can be accessed at https://dhsprogram.com/data/available-datasets.cfm.

## Abbreviations

AGYW: Adolescent girls and young women
DHS: Demographic and health survey
KDHS: Kenya demographic and health survey
LARC: Long-acting reversible contraceptives
SSA: Sub-Saharan Africa
TFR: Total fertility rate
